# Visualization of Organ-Specific Lymphatic Growth: An Efficient Approach to Labeling Molecular Markers in Cleared Tissues

**DOI:** 10.3390/ijms24065075

**Published:** 2023-03-07

**Authors:** Carolin Christ, Zoltán Jakus

**Affiliations:** Department of Physiology, Semmelweis University School of Medicine, 1094 Budapest, Hungary

**Keywords:** organ-specific lymphatic growth, lymphatic vessel growth, lymphatics, tissue clearing, CUBIC, primary lymphedema, *Flt4^kd/+^*, lymphatic dysfunction, molecular markers

## Abstract

Organ-specific lymphatics are essential for the maintenance of healthy organ function and lymphatic dysfunction can lead to the development of various diseases. However, the precise role of those lymphatic structures remains unknown, mainly due to inefficient visualization techniques. Here, we present an efficient approach to visualizing organ-specific lymphatic growth. We used a modified CUBIC protocol to clear mouse organs and combined it with whole-mount immunostaining to visualize lymphatic structures. We acquired images using upright, stereo and confocal microscopy and quantified them with AngioTool, a tool for the quantification of vascular networks. Using our approach, we then characterized the organ-specific lymphatic vasculature of the *Flt4^kd/+^* mouse model, showing symptoms of lymphatic dysfunction. Our approach enabled us to visualize the lymphatic vasculature of organs and to analyze and quantify structural changes. We detected morphologically altered lymphatic vessels in all investigated organs of *Flt4^kd/+^* mice, including the lungs, small intestine, heart and uterus, but no lymphatic structures in the skin. Quantifications showed that these mice have fewer and dilated lymphatic vessels in the small intestine and the lungs. Our results demonstrate that our approach can be used to investigate the importance of organ-specific lymphatics under both physiological and pathophysiological conditions.

## 1. Introduction

Lymphatic vessels have long been known to play a role in fluid homeostasis, immune cell trafficking and dietary lipid uptake. Recent studies have shown that lymphatics have additional organ-specific functions and that lymphatic dysfunction can lead to the development of disorders such as obesity, atherosclerosis, lung diseases, lymphedema and further metabolic diseases [1,2,3,4,5,6,7,8,9,10,11].

Lymphatic dysfunction can be caused by mutations of important regulators of lymphangiogenesis, such as *Flt4* [12,13]. A mouse model showing symptoms of lymphatic dysfunction is the *Flt4^kd/+^* strain, carrying a mutated *Flt4* allele which leads to the inactivation of the tyrosine kinase activity of vascular endothelial growth factor receptor-3 (VEGFR-3) [14]. These mice develop chylous ascites, show enlarged lymphatics in the intestinal tissue, are missing lymphatic vessels in the skin and suffer from swollen feet [14], but little is known about the lymphatic vasculature of other organs, mainly due to inefficient visualization techniques.

Despite obvious limitations, lymphatic vessels are still widely visualized by immunostaining of sections, although tissue sectioning is time-consuming and sections represent only small parts of a complex organ. Morphological changes of the lymphatic vasculature are difficult to capture and no information can be obtained about the length of lymphatic vessels, the number of lymphatic junctions or the total lymphatic area. Alternative visualization techniques are whole-mount immunostaining or lymphatic reporter mouse models, but the opacity of native tissue restricts light penetration and prevents sufficient imaging of the organ-specific lymphatic vasculature [15].

Tissue opacity is mainly caused by light scattering and absorption [15], both of which can be reduced by using tissue-clearing techniques. Tissue clearing aims to achieve an optically transparent tissue sample that can be used for image acquisition. Numerous protocols have been introduced, but not many protocols are suitable for the visualization of lymphatic vessels. Protocols such as Adipo-clear, CRISTAL, FASTClear, iDISCO and BABB contain hazardous organic solvents that often quench fluorescence signals, shrink organs and inevitably alter tissue morphology [16,17,18,19,20]. The CLARITY protocol utilizes an expensive solution called FocusClear™ in combination with a complex electrophoretic setup to clear organs [21]. WOBLI (whole organ blood and lymphatic vessel imaging) has been developed specifically for the visualization of lymphatic vessels, but clearing times are extremely long and the entire protocol can take up to 3 months to complete [22].

One of the most promising protocols is the CUBIC system (clear, unobstructed brain/body imaging cocktails and computational analysis), an efficient tissue-clearing protocol, developed to visualize whole mouse brains in single-cell resolution and later modified to clear whole mouse bodies [23,24,25]. CUBIC consists of two parts: the removal of lipids and pigments in the decolorization step followed by the matching of refraction indices in the RI-adjustment step, both of which result in a transparent tissue sample that can be used for imaging [23,24,25]. The latest protocols based on the original CUBIC protocol produce remarkable 3D images and use advanced quantification tools, but they require expensive light-sheet fluorescence microscopes (LSFMs) and specialized equipment as well as knowledge of machine learning and programming to quantify and analyze images [26,27,28]. The data volume of 3D images is vast and requires special computers and, more importantly, special knowledge for processing [24]. The analysis of LSFM images is time-consuming and the analysis of a single image can take hours and even days to complete [24,26].

Since these requirements discourage the majority of researchers from using tissue-clearing techniques on a regular basis, we developed an efficient approach to label molecular markers in cleared tissues in order to make tissue-clearing available to anyone. With our approach, we then characterized the organ-specific lymphatic vasculature of the *Flt4^kd/+^* mouse to investigate lymphatic growth under pathophysiological conditions.

## 2. Results

### 2.1. The Lymphatic Network Cannot Be Sufficiently Visualized with Conventional Visualization Methods

**Figure 1:**
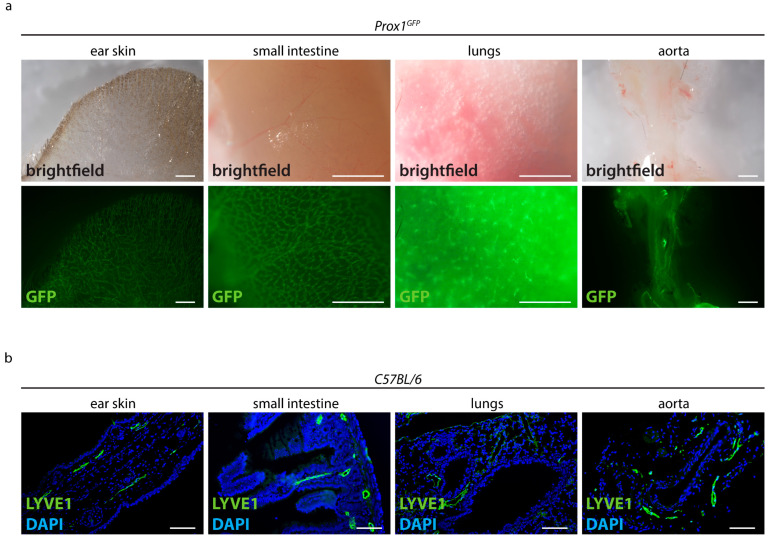
Visualization of the lymphatic vasculature with conventional techniques. (**a**) Detection of the native fluorescence of adult *Prox1^GFP^* lymphatic reporter mice. Brightfield images of various organs and the GFP signals of those samples are shown. Images were acquired by stereo microscopy, scale bar 1000 μm (n = 10 organs of 10 mice). (**b**) Visualization of lymphatic vessels in paraffin sections of adult *C57BL/6* mice stained with the lymphatic marker anti-LYVE1. Pictures were taken with upright microscopy, scale bar 100 μm (n = 10 organs of 10 mice). Representative images of the experiments are shown.

To test whether conventional visualization techniques can be used to visualize the organ-specific lymphatic vasculature, we stained tissue sections and used a lymphatic reporter mouse model (Figure 1). We first detected the native fluorescence in various organs of *Prox1^GFP^* lymphatic reporter mice expressing the green fluorescent protein (GFP) in lymphatic endothelial cells (Figure 1a). We could detect lymphatic vessels in the ear skin, small intestine, lungs and aorta, but due to the opacity of native tissue we could only visualize superficial lymphatic vessels.

We then performed paraffin-based histology on various organs of adult *C57BL/6* mice and stained sections with the lymphatic marker anti-LYVE1 (Figure 1b). Anti-LYVE1-staining showed that lymphatic vessels are present in the *villi intestinales* and in the submucosal layer of the intestinal tissue. We could detect lymphatic vessels around the bronchi of the lungs, in the dermis of the ear skin and around the aorta. Since sections only represent a limited part of an organ, it was impossible to assess the total length or area of lymphatic vessels or to efficiently quantify the organ-specific lymphatic vasculature of those organs.

### 2.2. Optimized Tissue-Clearing Protocol Clears Mouse Organs Quickly and Effectively

**Figure 2:**
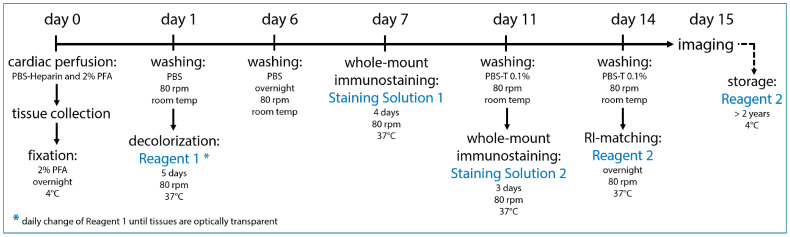
Optimized tissue-clearing protocol. Our optimized tissue-clearing protocol consists of an overnight fixation of the tissue sample, decolorization, whole-mount immunostaining with chosen antibodies and RI-matching before the sample is ready for imaging on day 15. Samples can be stored long-term at 4 °C in Reagent 2.

Our tissue-clearing protocol was 15 days long and consisted of the following main elements: a transcardial perfusion, tissue collection and post-fixation on day 0, decolorization for 5 days with Reagent 1, whole-mount immunostaining with the primary and secondary antibodies in Staining Solutions 1 and 2 and an RI-adjustment step in which the refraction indices of the cellular compartments were matched overnight in Reagent 2 (Figure 2).

**Figure 3:**
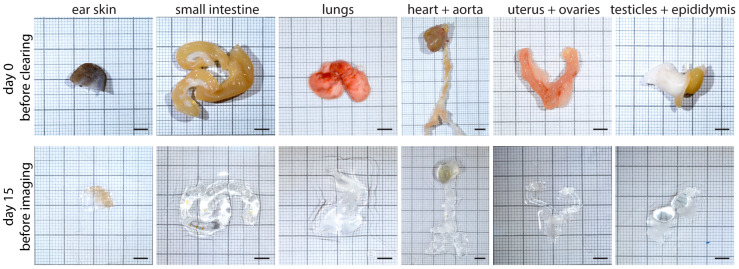
Tissue samples before and after the tissue-clearing protocol. Various organs of adult *C57BL/6* or *Prox1^GFP^* mice are shown on day 0 before the start of our protocol and on day 15 before imaging. Scale bar 5 mm (n = 5 organs of 5 mice). Representative images of the experiments are shown.

With this approach, we could successfully clear various mouse organs with everyday lab equipment. We tested our protocol with the ear skin, small intestine, lungs, heart with aorta, uterus with ovaries and testicles with epididymis and achieved a high level of transparency in all cases (Figure 3).

This tissue-clearing protocol is based on the previously described CUBIC protocol [23,24,25]. We modified the original protocol by omitting several steps, adjusting the incubation times and adding whole-mount immunostaining after the decolorization step.

### 2.3. Our Approach Enables Us to Visualize Organ-Specific Lymphatic Growth in Various Mouse Organs

**Figure 4:**
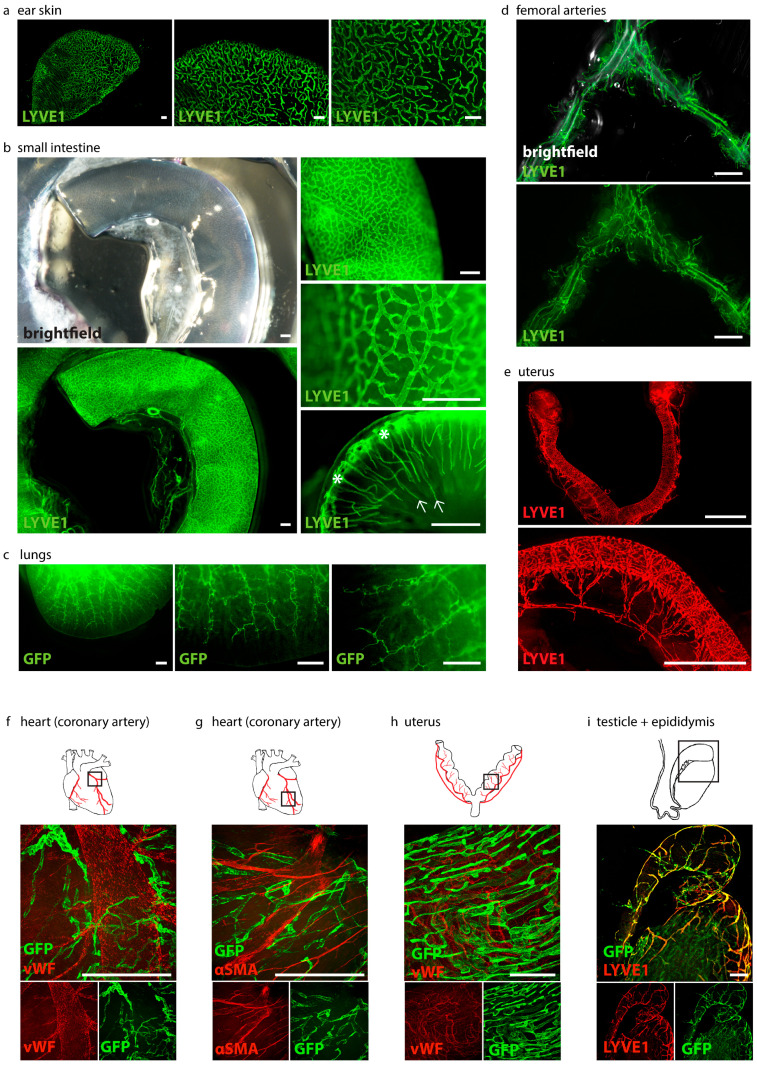
Visualization of organ-specific lymphatic vasculature. (**a**) Lymphatic vasculature of the ear skins of adult *C57BL/6* mice. Samples were stained with anti-LYVE1. Pictures were taken with a stereo microscope, scale bar 500 μm (n = 3 skin samples of 3 mice). (**b**) Lymphatic vasculature of the intestines of adult *C57BL/6* mice. Samples were stained with an anti-LYVE1 antibody. *Villi intestinales* are marked with arrows, the submucosal area is marked with asterisks. Pictures were taken with stereo microscopy, scale bar 500 μm (n = 3 small intestines of 3 mice). (**c**) Lymphatic vasculature of the lungs of adult *Prox1^GFP^* mice. Samples were stained with an anti-GFP antibody. Pictures were taken with stereo microscopy, scale bar 500 μm (n = 3 lungs of 3 mice). (**d**) Lymphatic vasculature of the femoral arteries of adult *C57BL/6* mice. Samples were stained with an anti-LYVE1 antibody. Pictures were taken with stereo microscopy, scale bar 5000 μm (n = 6 femoral arteries of 6 mice). (**e**) Lymphatic vasculature of the uterus of adult *C57BL/6* mice. Samples were stained with an anti-LYVE1 antibody. Pictures were taken with stereo microscopy, scale bar 2500 μm (n = 3 uteri of 3 mice). (**f**) Lymphatic vasculature of a coronary artery. Sample was stained with an anti-GFP antibody and anti-von Willebrand Factor (vWF). Pictures were taken with confocal microscopy, scale bar 500 μm (n = 1 coronary artery). (**g**) Lymphatic vasculature of the coronary artery of an adult *Prox1^GFP^* mouse. Sample was stained with an anti-GFP antibody and the smooth muscle marker anti-α-smooth muscle actin (αSMA). Pictures were taken with confocal microscopy, scale bar 500 μm (n = 1 coronary artery). (**h**) Lymphatic vasculature of the uteri of adult *Prox1^GFP^* mice. Samples were stained with anti-GFP and anti-vWF antibodies. Pictures were taken with confocal microscopy, scale bar 500 μm (n = 2 uteri of 2 mice). (**i**) Lymphatic vasculature of the testicle and epididymis of an adult *Prox1^GFP^* mouse. Sample was stained with anti-LYVE1 and with an anti-GFP antibody. Images were captured with confocal microscopy, scale bar 500 μm (n = 1 testicle with epididymis). Representative images of the experiments are shown.

To visualize organ-specific lymphatic growth in healthy animals, we tissue-cleared and stained organs of *C57BL/6* wild-type and *Prox1^GFP^* lymphatic reporter mice with various immunostainings (Figure 4).

We were able to visualize the organ-specific lymphatic vasculature of the ear skin of *C57BL/6* mice with an anti-LYVE1-staining (Figure 4a). Lymphatics are clearly visible in the images at higher magnifications.

We were also able to visualize the organ-specific lymphatic vasculature of the intestine of adult *C57BL/6* mice with an anti-LYVE1-staining (Figure 4b). Images at different magnifications show that the intestinal lymphatic vasculature can be visualized in great detail, even with a stereo microscope. Our approach allows us to visualize the morphology of the lymphatic network. Images of the lumen of the small intestine show the lacteals in the *villi intestinales* and the lymphatics in the submucosal region.

The organ-specific lymphatic vasculature of the lungs of *Prox1^GFP^* mice could be visualized with GFP staining (Figure 4c). The lymphatic network of the lungs is visible in different magnifications and the specific morphology of the lymphatic vessels is clearly visible. We then visualized the organ-specific lymphatic vasculature of the femoral arteries of *C57BL/6* mice with an anti-LYVE1 antibody (Figure 4d). Images show that the arteries are surrounded by a network of lymphatic vessels.

The lymphatic vasculature of the uteri of *C57BL/6* mice was visualized by an anti-LYVE1 staining (Figure 4e). Lymphatic vessels form an organized network surrounding the uterine horns and the cervix.

Visualization of cardiac lymphatics with an anti-GFP antibody and visualization of blood vessels with an anti-vWF (Figure 4f) or anti-αSMA antibody (Figure 4g) show the great potential of our tissue-clearing protocol. It enables us to label molecular markers with various antibodies in cleared tissues. Confocal microscopy enabled us to visualize the lymphatic vessels surrounding a coronary artery in great detail.

We then visualized the lymphatic vasculature of the uteri of *Prox1^GFP^* mice with anti-GFP and anti-vWF antibodies with confocal microscopy (Figure 4h). The image shows the lymphatic vessels and blood vessels of the uterine horn in higher magnification.

Anti-GFP and anti-LYVE1 staining helped us visualize the lymphatic vasculature of the testicle and epididymis of *Prox1^GFP^* mice (Figure 4i).

**Figure 5:**
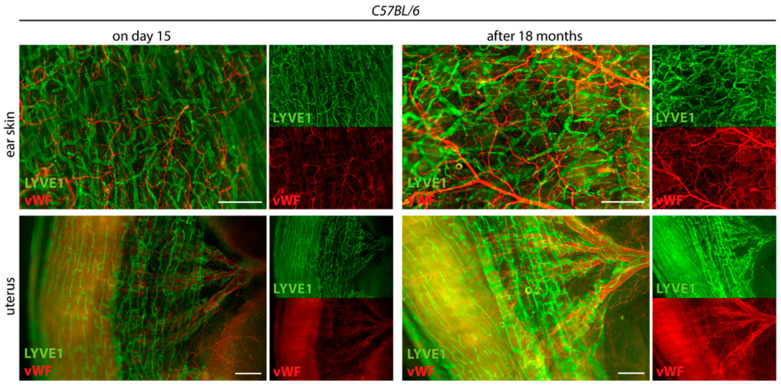
Long-term stability of the fluorescence signal. The tissue-cleared and stained ear skins and uteri of *C57BL/6* mice are shown on day 15 at the end of the tissue-clearing protocol and 18 months later. Samples were stained with the lymphatic marker anti-LYVE1 and anti-von Willebrand Factor (vWF). Samples were stored at 4 °C in Reagent 2. Images were acquired by stereo microscopy, scale bar 500 μm (n = 3 organs of 3 mice). Representative images of the experiments are shown.

Furthermore, we found that long-term storage of stained samples in Reagent 2 at 4 °C did not quench fluorescence signals. A strong, preserved fluorescent signal was still detectable 18 months after staining (Figure 5).

### 2.4. Lymphatic Structures Can Be Quantified with AngioTool, a User-Friendly Tool for the Analysis of Vascular Networks

**Figure 6:**
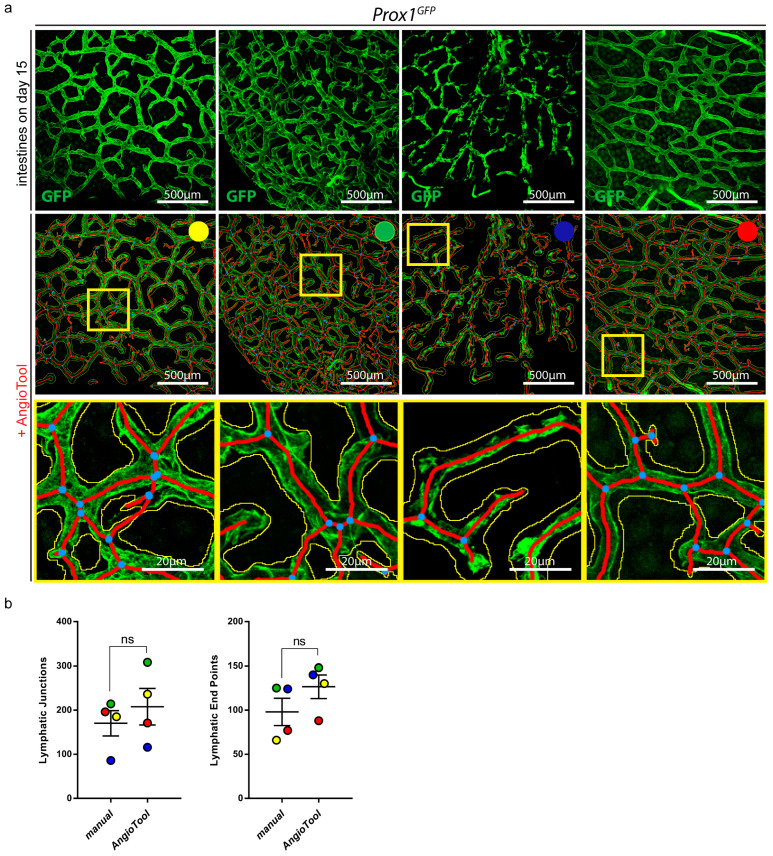
Quantification of organ-specific lymphatic vasculature. (**a**) Lymphatic vasculature of the small intestines of four *Prox1^GFP^* mice on day 15 after the tissue-clearing protocol. Samples were stained with an anti-GFP antibody. Images were acquired by confocal microscopy, scale bar 500 μm. Images were quantified with AngioTool, a user-friendly tool for the quantification of vasculature [29]. Every sample is marked with a specific color to analyze the results of the individual samples. Magnification of the images shows the quantification of AngioTool in detail: blue dots represent lymphatic junctions and red lines mark the length and the end points of lymphatic vessels. The yellow line surrounds the lymphatic area. Scale bar 20 μm. (**b**) Comparison of manual and AngioTool quantifications of the lymphatic junctions and lymphatic end points of the intestines shown in (**a**). Lymphatic junctions and lymphatic end points were quantified either manually with NIS-Elements imaging software or with AngioTool. Data are represented as mean ± SEM. Samples were statistically analyzed by a two-tailed, paired *t*-test; *p* = 0.2266 for lymphatic junctions, *p* = 0.0996 for lymphatic end points; ns = not significant; n = 4 small intestines of 4 mice.

To simplify the quantification process, we tested AngioTool, a free computational tool which was developed for the quantitative analysis of vascular networks [29]. We quantified the lymphatic junctions and lymphatic end points of four different confocal images of the small intestines of *Prox1^GFP^* mice with AngioTool (Figure 6a).

To evaluate whether the quantifications with AngioTool met our requirements, we manually quantified the lymphatic junctions and end points of the same images and compared the results (Figure 6b). To analyze the results of the individual images, we marked all images with a different color. We could not detect a significant difference between the manually quantified images and the images that were quantified with AngioTool.

### 2.5. Flt4^kd/+^ Mice Show Morphologically Altered Lymphatic Structures in All Investigated Organs

**Figure 7:**
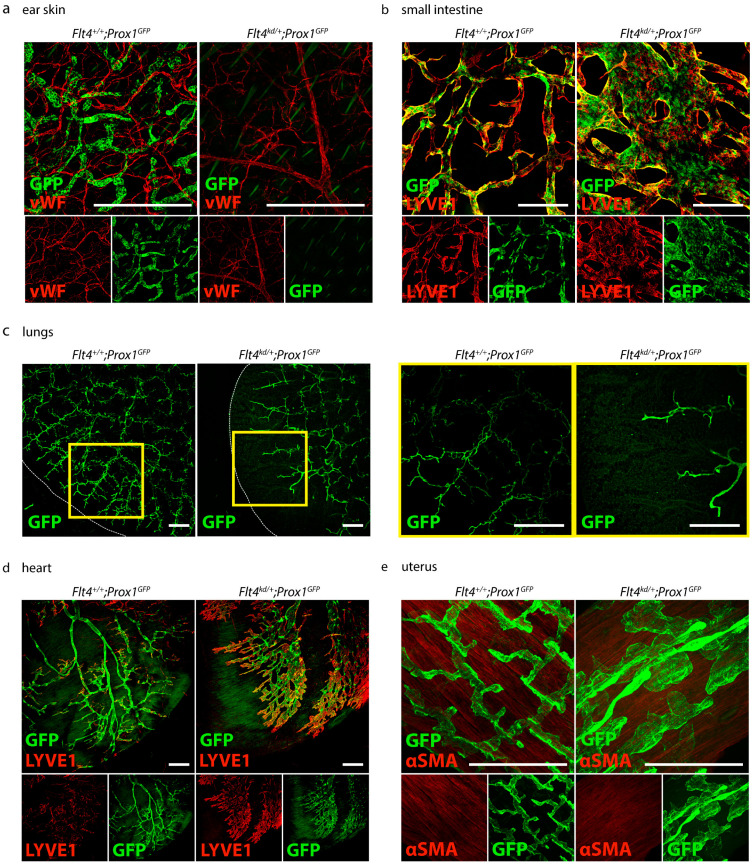
Visualization of the organ-specific lymphatic vasculature of *Flt4^kd/+^* mice. (**a**) Lymphatic vasculature of the ear skins of adult *Flt4^+/+^; Prox1^GFP^* and *Flt4^kd/+^; Prox1^GFP^* mice. Ears were stained with an anti-GFP antibody and anti-von Willebrand Factor (vWF). Images were acquired by confocal microscopy, scale bar 500 μm (n = 5 ears of 5 mice per group). (**b**) Lymphatic vasculature of the small intestines of adult *Flt4^+/+^; Prox1^GFP^* and *Flt4^kd/+^; Prox1^GFP^* mice. Intestines were stained with an anti-GFP antibody and anti-LYVE1. Pictures were taken with confocal microscopy, scale bar 500 μm (n = 6 small intestines of 6 mice per group). (**c**) Lymphatic vasculature of the lungs of adult *Flt4^+/+^; Prox1^GFP^* and *Flt4^kd/+^; Prox1^GFP^* mice. Lungs were stained with an anti-GFP antibody. Images were taken with a confocal microscope, scale bar 500 μm (n = 3–4 lungs of 3–4 mice per group). (**d**) Lymphatic vasculature of the hearts of adult *Flt4^+/+^; Prox1^GFP^* and *Flt4^kd/+^; Prox1^GFP^* mice. Hearts were stained with an anti-GFP antibody and anti-LYVE1. Images were taken with confocal microscopy, scale bar 500 μm (n = 3–4 hearts of 3–4 mice per group). (**e**) Lymphatic vasculature of the uteri of adult *Flt4^+/+^; Prox1^GFP^* and *Flt4^kd/+^; Prox1^GFP^* mice. Uteri were stained with an anti-GFP antibody and anti-alpha smooth muscle actin (αSMA). Images were acquired by confocal microscopy, scale bar 250 μm (n = 3–4 uteri of 3–4 mice per group). Representative images of the experiments are shown.

To visualize the organ-specific lymphatic growth in *Flt4^kd/+^* mice, we tissue-cleared and stained various organs of *Flt4^+/+^* and *Flt4^kd/+^* mice crossed with the *Prox1^GFP^* transgenic reporter strain (Figure 7).

First, we visualized the organ-specific lymphatic growth in the skin of *Flt4^+/+^; Prox1^GFP^* and *Flt4^kd/+^; Prox1^GFP^* mice with an anti-GFP antibody and anti-von Willebrand Factor (vWF) (Figure 7a). *Flt4^+/+^; Prox1^GFP^* mice show a normal blood and lymphatic vessel network in the skin. *Flt4^kd/+^; Prox1^GFP^* mice show normal blood vessels in the skin, but lymphatic vessels could not be detected.

We then visualized the lymphatic vasculature of the intestines of *Flt4^+/+^; Prox1^GFP^* and *Flt4^kd/+^; Prox1^GFP^* mice with an anti-GFP antibody and anti-LYVE1 (Figure 7b). *Flt4^+/+^; Prox1^GFP^* mice show normal and normal lymphatic vasculature in the small intestine. The GFP-positive nuclei of the lymphatic endothelial cells are clearly visible in the lymphatic structures. In comparison to their control littermates, *Flt4^kd/+^; Prox1^GFP^* mice show greatly enlarged and dilated lymphatic vessels in the intestine.

Next, we visualized the lymphatic vasculature of the lungs of *Flt4^+/+^; Prox1^GFP^* and *Flt4^kd/+^; Prox1^GFP^* mice with an anti-GFP antibody (Figure 7c). *Flt4^kd/+^; Prox1^GFP^* mice show fewer but dilated lymphatic structures in the lungs in comparison to their littermate control mice. The lymphatic vasculature did not reach the periphery of the lungs.

We then visualized the organ-specific lymphatic vasculature of the heart of *Flt4^+/+^; Prox1^GFP^* and *Flt4^kd/+^; Prox1^GFP^* mice with an anti-GFP antibody and the lymphatic marker anti-LYVE1 (Figure 7d). *Flt4^kd/+^; Prox1^GFP^* mice show extremely dilated lymphatic vessels in the heart.

Finally, we visualized the lymphatic vasculature of the uteri of *Flt4^+/+^; Prox1^GFP^* and *Flt4^kd/+^; Prox1^GFP^* mice with an anti-GFP antibody and the smooth muscle marker anti-alpha smooth muscle actin (αSMA) (Figure 7e) and found that *Flt4^kd/+^; Prox1^GFP^* mice show extremely dilated and morphologically altered lymphatic vessels in the uterus.

### 2.6. Morphological Changes of the Lymphatic Vasculature Can Be Detected with Our Approach but Not with Conventional Visualization Techniques

**Figure 8:**
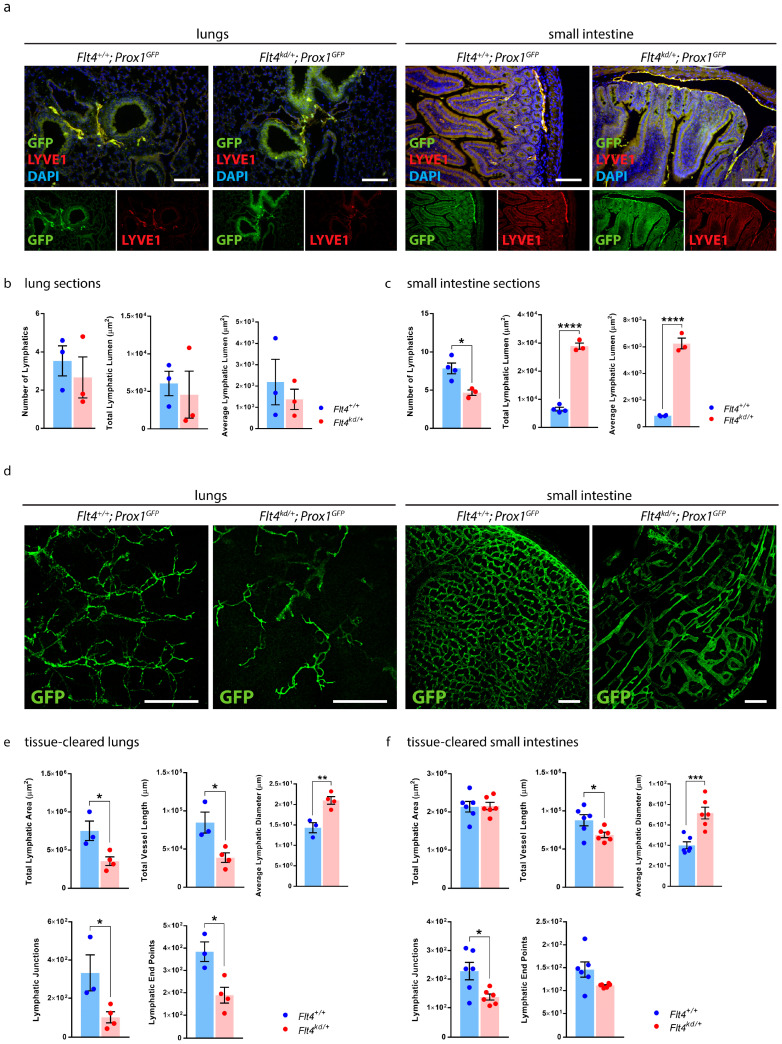
Quantification of the organ-specific lymphatic vasculature of *Flt4^kd/+^* Mice. (**a**) Visualization of the organ-specific lymphatic vasculature of the lungs and intestines of adult *Flt4^+/+^; Prox1^GFP^* and *Flt4^kd/+^; Prox1^GFP^* mice by immunostaining of sections. Sections were stained with an anti-GFP antibody and anti-LYVE1. Pictures were acquired by upright microscopy, scale bar 100 μm. Quantifications of the lung sections (**b**) and the small intestine sections (**c**) were performed manually using NIS-Elements imaging software and include the number of lymphatics, the total lymphatic lumen and the average lymphatic lumen per field of view. Data are represented as mean ± SEM. Samples were statistically analyzed by a two-tailed, unpaired *t*-test; *p* = 0.5502 for the number of lymphatics in the lung sections, *p* = 0.6925 for the total lymphatic lumen of the lung sections, *p* = 0.5262 for the average lymphatic lumen of the lung sections, *p* = 0.0152 for the number of lymphatics in the small intestine sections, *p* < 0.0001 for the total lymphatic lumen of the small intestine sections and *p* < 0.0001 for the average lymphatic lumen of the small intestine sections; * *p* < 0.05; **** *p* < 0.0001; n = 3–4 lungs or small intestines of 3–4 mice per group. (**d**) Tissue-cleared lungs and intestines of adult *Flt4^+/+^; Prox1^GFP^* and *Flt4^kd/+;^ Prox1^GFP^* mice. Samples were stained with an anti-GFP antibody. Images were acquired by confocal microscopy, scale bar 500 μm. Quantifications of the tissue-cleared lungs (**e**) and small intestines (**f**) were quantified with AngioTool and include the total lymphatic area, the total vessel length, lymphatic junctions and lymphatic end points per field of view. Lymphatic diameters were quantified manually using NIS-Elements. Data are represented as mean ± SEM. Samples were statistically analyzed by a two-tailed, unpaired *t*-test; *p* = 0.0256 for the total lymphatic area of tissue-cleared lungs, *p* = 0.0202 for the total vessel length of tissue-cleared lungs, *p* = 0.0068 for the average lymphatic diameter of tissue-cleared lungs, *p* = 0.0427 for the lymphatic junctions of tissue-cleared lungs, *p* = 0.0173 for the lymphatic end points of tissue-cleared lungs, *p* = 0.9156 for the total lymphatic area of tissue-cleared small intestines, *p* = 0.0377 for the total vessel length of tissue-cleared intestines, *p* = 0.0008 for the average lymphatic diameter of tissue-cleared small intestines, *p* = 0.0181 for the lymphatic junctions of tissue-cleared small intestines and *p* = 0.0631 for the lymphatic end points of tissue-cleared small intestines; * *p* < 0.05; ** *p* < 0.01; *** *p* < 0.001; n = 3–6 lungs or small intestines of 3–6 mice per group. Representative images of the experiments are shown.

To highlight the advantages of our protocol, we quantified images of lung and small intestine sections and images of tissue-cleared lungs and intestines (Figure 8).

We performed paraffin-based histology of the lungs and the small intestines of *Flt4^+/+^; Prox1^GFP^* and *Flt4^kd/+^; Prox1^GFP^* mice and stained them with an anti-GFP antibody and anti-LYVE1 (Figure 8a). We quantified the images of the lung sections manually with NIS-Elements imaging software (Figure 8b). Quantifications showed no significant differences regarding the number of lymphatics, the total lymphatic lumen and the average lymphatic lumen in the lungs between *Flt4^kd/+^; Prox1^GFP^* mice and their littermate control mice. We then quantified the images of the small intestine sections manually with NIS-Elements (Figure 8c). Quantifications showed a significant decrease in the number of intestinal lymphatics in *Flt4^kd/+^; Prox1^GFP^* mice. Total lymphatic lumen and the average lymphatic lumen were significantly increased in the small intestines of *Flt4^kd/+^; Prox1^GFP^* mice in comparison to the intestines of their littermate control mice.

To highlight the advantages of our approach, we tissue-cleared and stained lungs and small intestines of *Flt4^+/+^; Prox1^GFP^* and *Flt4^kd/+^; Prox1^GFP^* mice and acquired confocal images of the samples (Figure 8d).

Quantifications of the tissue-cleared lungs showed a decreased total lymphatic area, decreased total length of lymphatic vessels, fewer lymphatic junctions and fewer lymphatic end points in the lungs of *Flt4^kd/+^; Prox1^GFP^* mice in comparison to their controls, but a significant increase in the lymphatic diameter (Figure 8e). Our results suggest that *Flt4^kd/+^; Prox1^GFP^* mice have significantly fewer but dilated lymphatic vessels in the lungs.

Quantifications of the tissue-cleared small intestines showed no difference in the total lymphatic area but a significant decrease in the total length of lymphatic vessels, fewer lymphatic junctions and fewer lymphatic end points in the small intestines of *Flt4^kd/+^; Prox1^GFP^* mice in comparison to their controls, but a significant increase in the lymphatic diameter (Figure 8f). These results suggest that *Flt4^kd/+^; Prox1^GFP^* mice have fewer but dilated lymphatic vessels in the small intestine.

The results show that morphological changes in the lymphatic vasculature, especially changes in lymphatic parameters such as total lymphatic area, total length of lymphatic vessels, lymphatic junctions and end points can be detected effectively with our approach.

## 3. Discussion

In this study, we presented an efficient approach to visualize, analyze and quantify the organ-specific growth of lymphatic vessels. Conventional visualization techniques such as paraffin sections, reporter mouse models and whole-mount staining are not sufficient to visualize the lymphatic vasculature of organs due to the opacity of native tissue (Figure 1) [15]. Tissue-clearing techniques address this problem by clearing pigments and matching RI-indices, creating optically transparent tissue samples that can be used for imaging, but many of the protocols have limitations that make them unsuitable for the visualization of lymphatic vessels [16,18,19,21,22,26,30,31,32,33,34]. We decided to base our protocol on a non-toxic and efficient tissue-clearing protocol named CUBIC [23,24,25]. We modified the original protocol by omitting steps, changing incubation times and combining it with whole-mount immunostaining to be independent from reporter mouse models (Figure 2).

Our protocol enabled us to sufficiently clear various mouse organs in a reasonable time and without any visible shrinkage or damage to the tissue (Figure 3).

We showed that our protocol works with different fluorescent markers that allow us to visualize organ-specific lymphatic structures (Figure 4). We demonstrated that it can be used for double-staining with different antibody combinations and is suitable for the investigation of other structures of interest, such as blood vasculature (Figure 4f–h). With our approach, we then aimed to visualize and quantify the organ-specific lymphatic vasculature of the *Flt4^kd/+^* mouse, a mouse model showing symptoms of lymphatic dysfunction. The model was described nearly 20 years ago, but, aside from the lack of lymphatics in the skin and dilated intestinal lymphatics, little is known about the organ-specific lymphatic vasculature of this model [14].

We could confirm that these mice lack lymphatic vessels in the skin and that they have enlarged lymphatic vessels in the small intestine, as previously described [14]. Furthermore, we found that these mice have morphologically altered lymphatic vessels in the lungs, heart and uterus that have not yet been described (Figure 7).

The fluorescence signals of our samples remain stable for more than 18 months. Long-term stability of staining allows flexibility regarding the imaging of samples and even allows re-imaging of a sample after a long period of time if the focus of a study has changed (Figure 5).

In comparison to other CUBIC protocols [23,24,25,26], we could acquire images of great quality by stereo and confocal microscopy and did not need an expensive and complex LSFM (Figure 4, Figure 5, Figure 6, Figure 7 and Figure 8).

We focused on simplifying analysis and quantified our images using AngioTool (Figure 6 and Figure 8). AngioTool is a free and user-friendly computational tool specifically developed for the quantification of vascular structures [29]. It quantifies images in seconds by simply uploading them into the program. It allowed us to adjust parameters such as vessel diameter, intensity and particle size and to quantify vessel-relevant parameters such as total vessel area, total vessel length, number of vessel junctions and end points. Results generated with AngioTool showed no significant differences when compared to manual quantifications; therefore, we propose that AngioTool represents a suitable tool for quantifying images of lymphatic vasculature (Figure 6).

We then quantified sections of the lungs and small intestines of *Flt4^kd/+^* mice and compared them with quantifications of tissue-cleared lungs and small intestines (Figure 8). Based on the sections, we could quantify the number of lymphatic vessels, the total lymphatic lumen and the average lymphatic lumen. Results of the small intestine sections showed that lymphatic vessels are dilated in *Flt4^kd/+^* animals; results of the lung sections on the other hand were inconclusive. Based on the tissue-cleared samples, we were able to quantify more parameters, including lymphatic junctions, lymphatic end points and the total length of lymphatic vessels. Quantifications revealed that lungs and small intestines of *Flt4^kd/+^* animals have fewer lymphatic junctions and end points and that the total lymphatic area and length of lymphatic vessels is greatly reduced. The significantly increased lymphatic diameter shows that lymphatic vessels in the lungs and the small intestine are dilated, a sign of lymphatic malfunction. We showed that tissue-cleared samples allow a much better assessment of the lymphatic network of an organ than immunostaining of sections. Our protocol enabled us to visualize and quantify the differences between the organ-specific lymphatic vasculature of *Flt4^kd/+^* mice and their healthy littermate controls.

In this study, we demonstrated that our efficient protocol enabled us to visualize the lymphatic network of the studied organs, to detect differences and morphological alterations of lymphatic structures in mouse models and to quantify those differences. Many tissue-clearing protocols aim for high-resolution 3D-images of tissue-cleared organs but require expensive LSFM and previous knowledge of analyzing 3D-image data that are not readily available in every laboratory and therefore prevent the majority of researchers from using tissue-clearing techniques [18,21,26,30]. Here, we could provide an efficient protocol without the need for any special equipment or knowledge, making tissue-clearing possible for anyone. We showed that *Flt4^kd/+^* mice have morphologically altered organ-specific lymphatic vessels, which suggests that lymphatic vessels are important for the maintenance of healthy organ function. Further functional studies could help clarify the role of organ-specific lymphatic vessels and the impact of morphologically altered lymphatic vessels in the *Flt4^kd/+^* organs on the lymphedema phenotype.

## 4. Materials and Methods

### 4.1. Mouse Models

In this study, we used 3- to 5-month-old male and female *C57BL/6* and *Prox1^GFP^* BAC transgenic lymphatic reporter mice to visualize lymphatic vasculature [35]. *Prox1^GFP^* mice were maintained on a *C57BL/6* genetic background in heterozygous form and genotyped by a transgene specific PCR using 5′ -GAT GTG CCA TAA ATC CCA GAG CCT AT−3′ forward and 5′-GGT CGG GGT AGC GGC TGA A−3′ reverse primers. Additionally, we used 3- to 5-month-old *Flt4^kd/+^* mice, a previously described model for primary lymphedema, and their littermate control mice [14]. Mice carrying the kinase-dead *Flt4* allele (MRC Harwell, UK) were maintained on a NMRI background [14]. The *Flt4* point mutant allele was genotyped using 5′-CTGGCTGAGTCCCTAACTCG-3′ forward and 5’-CGGGGTCTTTGTAGATGTCC-3′ reverse primers, followed by a restriction enzyme digestion with BglII, as previously described [9]. Furthermore, *Flt4^+/+^* and *Flt4^kd/+^* mice were crossed with the *Prox1^GFP^* reporter strain, resulting in *Flt4^+/+^; Prox1^GFP^* and *Flt4^kd/+^; Prox1^GFP^* animals. All animals were housed under a 12/12 h light/dark cycle with unrestricted access to food and water. All procedures were carried out according to the Animal Experimentation Review Board of Semmelweis University and the Government Office for Pest County, Hungary.

### 4.2. Paraffin-Based Histology and Immunostaining of Sections

Mice were deeply anaesthetized by an intraperitoneal injection of 2.5% 2,2,2-Tribromoethanol (Sigma-Aldrich, T48402, St. Louis, MO, USA) and transcardially perfused with 10mL ice-cold phosphate-buffered saline (PBS)-Heparin (Teva Pharmaceuticals, Tel Aviv, Israel) (5000 IU/mL) followed by 10mL freshly prepared 4% paraformaldehyde (PFA) (Sigma-Aldrich, P6148, St. Louis, MO, USA). Tissue samples were collected and fixed overnight in 4% PFA at 4 °C, washed with PBS, dehydrated and embedded in paraffin using an embedding station (Leica, EG1150H, Wetzlar, Germany). A microtome (Thermo Fisher Scientific, HM340E, Waltham, MA, USA) was used to generate 7μm sections which were stained with the lymphatic marker goat-anti-LYVE1 (Bio-Techne, AF2125, Minneapolis, MN, USA) in a dilution of 1:100 and anti-goat secondary antibody conjugated to Alexa Fluor 488 (Thermo Fisher Scientific, A11055, Waltham, MA, USA) in a dilution of 1:250. Nuclear staining with 4′,6-Diamidino-2-phenylidole (DAPI) (Vector Laboratories, Inc., H-1200, Burlingame, CA, USA) helped to visualize the gross morphology of the section. Images were taken with an upright microscope (Nikon Instruments, ECLIPSE Ni-U, Tokyo, Japan) connected to a camera (Nikon Instruments, DS-Ri2, Tokyo, Japan).

### 4.3. Detection and Imaging of Native Fluorescence

Mice were deeply anaesthetized by an intraperitoneal injection of 2.5% 2,2,2-Tribromoethanol followed by cardiac perfusion with 10 mL ice-cold PBS-Heparin (5000 IU/mL). Tissue samples were collected, washed with PBS and visualized with a stereo microscope (Nikon Instruments, SMZ25, Tokyo, Japan) connected to a camera (Nikon Instruments, DS-Ri2, Tokyo, Japan).

### 4.4. Tissue Clearing and Whole-Mount Immunostaining

#### 4.4.1. Cardiac Perfusion and Tissue Collection

Mice were deeply anaesthetized by an intraperitoneal injection of 2.5% 2,2,2-Tribromoethanol on day 0 and transcardially perfused with 10 mL ice-cold PBS-Heparin (5000 IU/mL) followed by 10 mL freshly prepared 2% PFA. Tissue samples were collected and fixed overnight in 2% PFA at 4 °C.

#### 4.4.2. Decolorization and Delipidation

Our tissue-clearing protocol is based on the previously published CUBIC protocol [23,24,25].

On day 1, the fixed samples were washed with PBS and immersed in Reagent 1 (25 wt% urea (Sigma-Aldrich, U5378, St. Louis, MO, USA), 25 wt% N, N, N′, N′-tetrakis (2-hydroxypropyl) ethylenediamine (Sigma-Aldrich, 122262, St. Louis, MO, USA) and 15 wt% Triton™ X-100 (Sigma-Aldrich, X100, St. Louis, MO, USA) in dest. H_2_O). Incubation in Reagent 1 lasted for 5 days at 37 °C and 80 rpm. Reagent 1 was changed daily. On day 6, the transparent tissues were washed twice with PBS and rehydrated overnight in PBS, room temperature, 80 rpm.

#### 4.4.3. Whole-Mount Immunostaining

On day 7, the rehydrated tissue samples were stained for 4 days with the primary antibody(s) in Staining Solution 1 (serum (10%), sodium azide (0.2%), Tween^®^ 20 (Sigma-Aldrich, P1379, St. Louis, MO, USA) (0.1%) in PBS with primary antibody(s) in the following dilutions: goat-anti-LYVE1 (Bio-Techne, AF2125, Minneapolis, MN, USA) in a dilution of 1:650, rabbit-anti-GFP (LifeTechnologies, A11122, Carlsbad, CA, USA) in a dilution of 1:500 and mouse-anti-αSMA (Sigma-Aldrich, A5228, St. Louis, MO, USA) in a dilution of 1:500) at 37 °C, 80 rpm. On day 11, the samples were washed with PBS-Tween^®^ 20 (0.1%) for 2 h at room temperature, 80 rpm, and then stained for 3 days with the secondary antibody(s) in Staining Solution 2 (serum (2%), sodium azide (0.2%), PBS-Tween^®^ 20 (0.1%) in PBS with secondary antibody(s) in the following dilutions: donkey-anti-goat Alexa Fluor 488 (Thermo Fisher Scientific, A11055, Waltham, MA, USA) in a dilution of 1:3000, donkey-anti-goat Alexa Fluor 568 (Thermo Fisher Scientific, A11057, Waltham, MA, USA) in a dilution of 1:3000, donkey-anti-rabbit Alexa Fluor 488 (Thermo Fisher Scientific, A21206, Waltham, MA, USA) in a dilution of 1:3000 and donkey-anti-mouse Alexa Fluor 568 (Thermo Fisher Scientific, A10037, Waltham, MA, USA) in a dilution of 1:3000) at 37 °C, 80 rpm (tubes were wrapped in tinfoil to prevent degradation of the fluorophores during the incubation process). On day 14, stained tissues were washed for 2 h with PBS-Tween^®^ 20 (0.1%) at room temperature, 80 rpm. Control stainings have been performed for all antibodies used in this study.

#### 4.4.4. Adjustment of the Refraction Indices (RI-Adjustment)

The stained samples were then incubated overnight in Reagent 2 (50 wt% sucrose (Sigma-Aldrich, S7903, St. Louis, MO, USA), 25 wt% urea, 10 wt% triethanolamine (Sigma-Aldrich, 90279, St. Louis, MO, USA), 0.1 wt% Triton™ X-100 in dest. H_2_O) at 37 °C, 80 rpm. On day 15, samples were ready for imaging and were transferred to 4 °C for long-term storage.

#### 4.4.5. Microscopic Imaging and Processing

On day 15, samples were imaged with a stereo microscope connected to a camera or a confocal microscope (Nikon Instruments, A1 HD25, Tokyo, Japan) connected to a confocal scanner unit (Yokogawa Electric Corporation, CSU-W1, Tokyo, Japan). Images were processed and analyzed using NIS-Elements imaging software (Nikon Instruments, version BR 4.60.00).

### 4.5. Manual Quantification of Vascular Structures

Manual quantifications were performed using NIS-Elements. For the quantification of sections, lymphatic number, total lymphatic lumen and average lymphatic lumen of 5 fields of view (20× magnification) were quantified per mouse. For the quantification of the lymphatic diameter of tissue-cleared lungs and intestines, a minimum of 40 lymphatic vessels (10× magnification) were measured per mouse.

### 4.6. Quantification of Vascular Structures with AngioTool

Total lymphatic area, total length of lymphatic vessels, lymphatic junctions and lymphatic end points were quantified with AngioTool, a free computational tool that has been developed for the quantitative analysis of vascular networks [29]. After uploading the images to AngioTool, we adjusted parameters such as the vessel diameter and intensity and removed small particles from the calculation. All images were quantified using the same parameters.

### 4.7. Data Presentation and Statistical Analysis

Representative images of the experiments are shown. Data were processed and statistically analyzed using GraphPad Prism (version 7.03) and Excel (Microsoft, version 2018).

## Data Availability

Datasets for this study are available from the corresponding author upon reasonable request.
